# The Mediating Role of Positive Attitudes on the Relationship Between Esports Gaming Hours and Psychological Well-Being During the COVID-19 Pandemic

**DOI:** 10.7759/cureus.36334

**Published:** 2023-03-18

**Authors:** Dan Shan, ZhiHao Dai, FenFen Ge, Yanyi Zhang, YuanDian Zheng, XiaoYi Gao, JunChu Han

**Affiliations:** 1 Department of Biobehavioral Science, Teachers College, Columbia University, New York City, USA; 2 School of Medicine, Royal College of Surgeons, Ireland, Dublin, IRL; 3 Faculty of Medicine, University of Iceland, Reykjavik, ISL; 4 Faculty of Medicine and Health, The University of Sydney, Sydney, AUS; 5 College of Osteopathic Medicine, Kansas City University, Kansas City, USA; 6 School of Medicine, Saint Louis University, St. Louis, USA; 7 Department of Biobehavioral Sciences, Teachers College, Columbia University, New York City, USA

**Keywords:** mental health, gaming hour, electronic sports game, covid-19, attitude

## Abstract

Electronic sports game (esports) gaming has seen a surge in popularity, especially during the coronavirus disease 2019 (COVID-19) pandemic, with more young people turning to it as an alternative to physical activities. However, the impact of esports gaming on mental health is a matter of concern. Previous studies have produced inconsistent findings on the relationship between gaming hours and mental health, and the moderating factors involved remain unexplored. This study aimed to investigate the moderating effect of participants' subjective attitudes toward esports gaming on the relationship between daily gaming hours and psychological well-being (PWB) among Chinese young adults during the COVID-19 lockdown. A nationwide online survey was conducted on 550 Chinese young adults using the Credamo platform. Ryff's Psychological Well-Being Scales (42-Item version) were used to assess PWB levels. The analysis included 453 participants. Gaming hours were negatively correlated with PWB scores. However, when considering the moderating effect of subjective attitudes, the association between gaming hours and PWB scores was largely positive. Our study suggests that subjective attitudes toward esports gaming outweigh gaming hours in promoting personal psychological well-being. We propose practical recommendations for healthy esports participation patterns that prioritize positive attitudes, especially in similar future scenarios like COVID-19. Our findings may inform future psychological intervention and research in the esports domain.

## Introduction

In light of stay-at-home and social-distancing measures, electronic sports game (esports), which takes place in the virtual domain, has experienced a surge in participation during the coronavirus disease 2019 (COVID-19) pandemic [1,2]. While it is well-established that online gaming can lead to addiction [3-6], the pandemic has presented a unique opportunity for esports to have positive effects on individuals [7,8]. With lockdown restrictions severely limiting personal socialization, esports online gaming, particularly the communication element through team cooperation, has provided alternative social channels and fostered interpersonal connections, ultimately enhancing personal mental health [9-12]. However, due to the highly competitive nature of esports gaming, adverse effects on mental health may be caused by certain conditions such as increased levels of stress markers [13].

Previous studies have held opposite opinions on the impact of online gaming on mental health [3-8], indicating that something may moderate the relationship between gaming hours and well-being, ultimately leading to different outcomes. The subjective attitudes of players toward online gaming have not been adequately explored, particularly in the context of esports gaming, despite the powerful influence of subjective attitudes on individual behaviors [8,14,15]. Given the notable differences between the general types of online games and esports games, it is essential to investigate the role of subjective attitudes on the relationship between gaming hours and personal psychological well-being (PWB), depending on different genres of games [16].

Thus, the primary purpose of this study was to investigate the moderating effect of subjective attitudes toward esports gaming on average daily gaming hours in collectively influencing PWB in Chinese young adults during the COVID-19 lockdown in China, thereby enriching the research on the effects of esports gaming on individuals’ mental health, and discussing potentially particular ways of esports gaming that may be more beneficial for those in lockdown or similar situations.

The highly competitive nature of esports might negatively impact individual mental health by keeping players constantly on edge, and players often value the result of winning or losing the game [13]. Therefore, subjective attitudes toward esports gaming may moderate the relationship between daily gaming hours and psychological well-being. Accordingly, the following hypotheses were proposed: 1) Increased average daily gaming hours as a separate variable might be negatively associated with personal psychological well-being; 2) Subjective attitudes toward esports gaming moderate the relationship between daily gaming hours and psychological well-being.

## Materials and methods

Overview

We conducted a questionnaire survey targeting Chinese young adults aged 18 to 25 years using Credamo, a professional online survey platform, from September 1, 2020, to September 30, 2020. As a bilingual digital data collection platform with both Chinese and English interfaces, Credamo provides functionality comparable to international online sampling platforms, such as Qualtrics and MTurk, facilitating efficient and versatile data acquisition across diverse populations. The use of human data from the surveys was conducted in accordance with the principles of the Declaration of Helsinki (as revised in 2013). Prior to completing the survey, all participants received a full explanation of the survey's purpose and were asked to confirm online informed consent. Furthermore, all data were collected anonymously through Credamo using continuous identifier numbers to distinguish participants, rather than recording their names or other sensitive information.

To ensure the survey quality, we used two attention-check questions at different points in the survey. We also manually checked the time taken for completing each survey and the IP address of responders in case of multiple responses from the same responder. Additionally, participants were informed to answer the questionnaire truthfully under the personal-psychological status developed explicitly during the most severe periods of the pandemic outbreak in China, and only those who had played targeted esports games listed on the first page were allowed to complete the questionnaire, including League of Legends (LOL), DOTA2, Honor of Kings, CS: GO, CrossFire (CF), Overwatch (OW), PUBG, and PUBG Mobile, which are currently the most relatively popular esports games among young adults in China [17].

Questionnaire contents

The questionnaire mainly contained the following information: Demographic information; Esports engagement during the pandemic, including the platform where the participants played esports games and average daily gaming hours; Subjective attitudes toward esports gaming in influencing psychological well-being (i.e., ‘Do you think playing esports games can positively improve psychological well-being during the pandemic?); Ryff’s Psychological Well-Being Scales (PWB), 42-Item version [18]; Whether the participants experienced any personal circumstances unrelated to esports gaming that led to a significant deterioration in their mental status (See Appendices I & II).

A total of 550 young adults were recruited and surveyed via the Credamo platform, and after the exclusion of participants who failed attention-check questions (e.g., responded wrongly to the instruction “please choose the answer Pink”), took less than 200 seconds to complete the survey, provided inconsistent or contradictory responses to questionnaire questions, or experienced personal COVID-19-related conditions that significantly affected their mental state, a valid sample of 453 participants were analyzed collectively (261 females and 192 males; mean age = 21.51 years, Standard deviation (SD) = 1.93 years; age range: 18-25 years). The effective response rate was 82.4%.

Psychological well-being assessment tool

The psychological well-being of the participants was assessed using the 42-item version of Ryff's PWB Scale, which measures six dimensions of well-being and happiness: Autonomy (AU), Environmental mastery (EM), Personal growth (PG), Positive relations with others (PR), Purpose in life (PL), and Self-acceptance (SA). Participants were asked to choose the degree to which they agreed or disagreed with each statement, on a scale of 1 to 6, with 1 indicating "strongly disagree" and 6 indicating "strongly agree". In this study, 20 negatively worded items were reversely scored before analysis so that a higher final total well-being score, obtained by summing all six subscales, indicated a higher level of psychological well-being. Meanwhile, a higher score in a particular subscale indicated a higher level of psychological well-being state in a specific dimension [19-21]. Cronbach’s alpha coefficient for the total scale was 0.92, suggesting excellent overall internal consistency.

Statistical analysis

We performed all statistical analyses using the software program SPSS version 26.0 (IBM Corp., Armonk, NY), except for the data cleaning process, which included the detection and removal of invalid or missing data completed on the Credamo data platform. We performed reliability tests for Ryff's PWB Scale and each subscale, using Cronbach’s alpha coefficient as a measure of internal consistency (α > 0.70 regarded as acceptable). We used parametric tests to compare mean differences. To analyze the moderating effect of participants' subjective attitudes toward playing esports games on gaming hours in collectively determining the PWB score as an outcome, we used hierarchical multiple regression. We entered gender, age, identity, and exercise habit in the first step to control for potential confounding variables. In the second step, we included variables related to esports gaming engagement during the pandemic. Finally, in the third step, we entered participants’ subjective attitudes as a moderator variable for average daily gaming hours.

## Results

Sample characteristics

Of the 550 individuals who enrolled in the survey, 453 (82.4%) were included in the analysis after data cleaning. Table 1 presents relevant descriptive statistics.

**Table 1:** Sample description

Variables		n (%)
Total		453 (100.00)
Demographic characteristics		
Gender		
Male		192 (42.38)
Female		261 (57.62)
Age (mean 21.5±1.9 years)		
Identity of participants		
Undergraduate		363 (80.13)
Post-graduate		46 (10.15)
Employee		44 (9.72)
Indoor exercise habit (over 20 mins per day)		
Yes		171 (37.75)
No		282 (62.25)
Esports games engagement characteristics		
Had been a fan of esports games before the pandemic		
Yes		309 (68.21)
No		144 (31.79)
Gaming platform		
Based on personal computer	152 (33.55)	
League of Legends	31 (6.84)	
DOTA2	43 (9.49)	
CS: GO	48 (10.60)	
CrossFire	5 (1.10)	
Overwatch	7 (1.55)	
PUBG	18 (3.97)	
Based on mobile phone	301 (66.45)	
Honor of Kings	213 (47.02)	
PUBG Mobile (i.e., Game for peace)	88 (19.43)	
Average time spent daily on esports games		
Less than 1h		55 (12.1)
1 to 3 h		215 (47.5)
3 to 5 h		137 (30.2)
5 to 7 h		29 (6.4)
Over 7 h		17 (3.8)
Subjective attitudes on role of playing esports games in affecting psychological well-being		
Has potential negative impacts		3 (0.66)
Has no positive or negative impact		14 (3.09)
Be able to slightly improve		189 (41.72)
Be able to moderately improve		168 (37.09)
Be able to strongly improve		79 (17.44)

Mean comparison of psychological well-being

Table 2 shows the mean differences in psychological well-being. Our results indicated a statistically significant difference in terms of identity (F = 4.04, p < 0.05) and a significant difference with respect to having an indoor exercise/physical activity habit (t = -4.19, p < 0.001). Participants with a habit of indoor exercise/physical activity had higher scores of psychological well-being than those without such experience. There was no significant difference based on other parameters (all p > 0.05).

**Table 2:** Psychological well-being (PWB scores) of participants (n = 453) *. indicates statistically significant at p < 0.05 level (2-tailed) **. indicates statistically significant at p < 0.01 level (2-tailed) ***. indicates statistically significant at p < 0.001 level (2-tailed)

Variables	Psychological Well-Being (total score)			
	Means (SD)	(95%CI)	t/F	p
Demographic characteristics				
Gender			-0.68	0.495
Male	164.38 (21.07)			
Female	162.95 (22.62)			
Identity			4.04	0.018^*^
Undergraduate	162.81 (22.05)	(160.54, 165.09)		
Post-graduate	172.04 (20.55)	(165.94, 178.15)		
Employee	160.79 (21.12)	(154.37, 167.21)		
Regular exercise habit			-4.19	0.000^***^
Yes	169.02 (20.42)			
No	160.25 (22.24)			
Esports games engagement				
A fan of esports games before the pandemic			-0.02	0.984
Yes	163.57 (22.14)			
No	163.53 (21.66)			
Gaming platform			-1.78	0.077
PC	160.99 (21.28)			
Mobile	164.86 (22.22)			
Daily gaming hours			1.15	0.333
Less than 1h	165.47 (24.08)	(158.96, 171.98)		
1 to 3 h	162.27 (21.42)	(159.39, 165.15)		
3 to 5 h	165.79 (21.17)	(162.22, 169.37)		
5 to 7 h	163.27 (25.20)	(153.68, 172.86)		
Over 7 h	156.00 (21.67)	(144.85, 167.14)		

Hierarchical multiple linear regression analysis

Table 3 presents the values of all factors relevant to the hierarchical multiple linear regression analyses while Table 4 shows the results. In the hierarchical multiple linear regression models, we did not find any multicollinearity problem in all three steps of the models. The correlation between psychological well-being scores and indoor exercise/physical activity habits was strongly statistically significant and slightly positive in all three steps of the model (all β > 0.15, p < 0.001). In the third step of the model, we found that mobile gamers tended to have slightly higher PWB scores as compared to PC gamers (β = 0.18, p < 0.01). We also found that average daily gaming hours were negatively correlated with PWB scores (β = -0.37, p < 0.001), which was in line with our first hypothesis. However, when considering the moderating effect of subjective attitudes toward esports gaming on gaming hours in collectively influencing psychological well-being, we found that the correlation between daily gaming hours and PWB scores turned in the opposite direction to a large extent (β = 0.40, p < 0.001), which was consistent with our second hypothesis. Although all factors in the hierarchical model did not show significant contribution in predicting the PWB scores (R2 = 0.09, ΔR2 = 0.03, ΔF = 16.16, p < 0.001), this could be understandable because playing esports games could not determine personal psychological well-being to a large degree in any way, and directly predicting one's psychological well-being just based on esports gaming was not the main purpose of this study.

**Table 3:** Factors’ values assigned in the hierarchical multiple linear regression analysis Notes: a represents the subjective attitudes of participants toward playing esports games in influencing personal psychological well-being.

Variables	Values
Gender	1 = Female, 2 = Male
Age	Primary Value
Identity of participants	1 = Undergraduate, 2 = Postgraduate, 3 = Employee
Indoor exercise habit	1 = No, 2 = Yes
A fan of games pre-pandemic	1 = No, 2 = Yes
Gaming platform	1 = Based on personal computers, 2 = Based on mobile phones
Average daily gaming hours	1 = <1 hour, 2 = 1-3 hours, 3 = 3-5 hours, 4 = 5-7 hours, 5 = >7 hours
^a^Subjective attitudes	1 = Negative impact, 2 = Neutral impact, 3 = Slightly positive impact, 4 = Moderately positive impact, 5 = Strongly positive impact
Psychological well-being scores	Primary Value

**Table 4:** Hierarchical regression analyses predicting psychological well-being (PWB). Abbreviations: AFGPP: a fan of games pre-pandemic, DGH: daily gaming hours, GP: gaming platform, IEH: indoor exercise/physical activity habit, SA: subjective attitudes aSA represents the subjective attitudes of participants toward playing esports games in influencing personal psychological well-being Betas were standardized. *P < 0.05; **P < 0.01; ***P < 0.001

Predictors	R^2^	ΔR^2^	ΔF	Stand β
Step 1	0.04^**^	0.04^**^	4.73^**^	
Gender				0.17
Age				-0.00
Identity				-0.01
IEH				0.20^***^
Step 2	0.06^***^	0.02^*^	3.65^*^	
Gender				0.10
Age				-0.00
Identity				0.01
IEH				0.19^***^
DGH				-0.03
GP				0.15^*^
Step 3	0.09^***^	0.03^**^	16.16^***^	
Gender				0.10
Age				0.00
Identity				-0.02
IEH				0.18^***^
DGH				-0.37^***^
GP				0.18^**^
^a^SA x DGH				0.40^***^

## Discussion

The results of the study initially suggested that increasing daily gaming hours was negatively correlated with PWB when controlled for other variables. This finding is consistent with previous studies [22-25]; nonetheless, contrary to two recent studies [26,27]. When the subjective attitude toward esports gaming is taken into account, the negative association no longer exists, suggesting that a positive mindset is a mitigating factor that modulates the experience and can contribute positively toward mental health. In addition, those who regularly exercised performed better in PWB scores as compared to those who did not exercise, which is consistent with the literature on exercise and mental health [28-30]. The study also found that using mobile devices is better than PC gaming for mental health, possibly because games on mobile devices are generally more relaxing, and people tend to spend less time on them. Further work should be done to analyze the time people spend on average gaming on each device to explore this further.

Previous studies on this subject are conflicting. Several previous studies have suggested that gaming could have negative effects on mental health [22-24]. These results need to be understood in the context of pre-pandemic and pandemic lockdown measures. More recent studies looking at gaming hours suggested a more positive relationship [26,27], especially in the COVID-19 lockdown context [26], which can be consistent with our findings after the subjective attitudes of participants toward esports gaming were considered. Johannes et al. (2021) demonstrated that participants who found the game enjoyable were more likely to gain a positive impact on their mental health [27], noting the importance of subjective attitudes toward the game itself. Moreover, the game the study chose was Plants vs. Zombies, which is not characterized as an esports game and is generally more relaxing, requiring less emotional and time investment per round. This may imply that creating a relaxed environment during gaming may be conducive to a positive emotional experience and thus personal well-being. Hence, generating positive feelings during gaming could possibly be more influential than the actual amount of time spent on video games in boosting psychological well-being. Additionally, despite Barr et al. suggesting that online gaming promoted the well-being of the overwhelming majority of participants during the pandemic, they collected relevant data by directly asking about participants’ feelings rather than objectively collecting them from a professional psychological scale [26]. As a result, the results may be relatively less reliable. In fact, the question from their survey ‘Do you feel that playing video games has had any impact on your well-being during the COVID-19 outbreak? The impact may be positive or negative’ was very similar to the question of subjective beliefs conducted in our questionnaire survey. Although most participants in our study thought that esports gaming was capable of improving psychological well-being to different extents, our initial finding suggesting that gaming hours were negatively associated with well-being was inconsistent with the positive beliefs of those participants. Consequently, the finding in Barr et al.’s study had limited valuable reference to evaluating the role of online gaming on well-being.

Esports gaming has emerged as a valuable social outlet during the COVID-19 pandemic, as it offers opportunities for social interaction and can have positive effects on psychological well-being [9-11,31,32]. Due to the closure of many public entertainment options and activities, esports gaming has become an attractive option for people who desire social connections. During gameplay, players can express themselves comfortably and safely in ways that may not be possible in real life due to appearance, personality, and other factors [33]. The resulting social connections can either be newly established or consolidated, helping release neurotransmitters such as dopamine and oxytocin, which can enhance emotional functions and promote personal well-being [34-36]. However, while esports gaming has many benefits, it can also lead to emotional dissatisfaction due to its highly competitive nature, in which winning and losing can result in negative emotions [37]. Hence, nurturing a positive outlook on esports gaming, incorporating the practical suggestions outlined in after-mentioned Table 5, is vital from this perspective as well.

The present study provides evidence for the significance of individuals' subjective beliefs about esports gaming in modulating their psychological well-being, highlighting the close interaction between attitudes and behaviors [16,17]. Conflicting attitudes and behaviors can lead to cognitive dissonance, prompting individuals to modify their behaviors to align with their attitudes [16]. Desirable behaviors can generate feedback, strengthening or shifting pre-existing beliefs [17]. In the case of esports gaming, participants who hold optimistic attitudes are more likely to generate positive feelings, deal positively with difficulties, and view failures as an opportunity for reflection and improvement, resulting in genuine enjoyment. These positive sentiments can then further reinforce their beliefs, resulting in a positive feedback loop and increased psychological well-being. Conversely, individuals with negative attitudes are likely to experience the opposite effects, regardless of the amount of time spent on esports games. Thus, this study helps bridge the contradictory findings about the impacts of gaming hours on psychological well-being, suggesting that positive attitudes toward esports gaming are essential for improving psychological well-being.

However, it is important to note that esports gaming alone cannot predict an individual's overall mental well-being, and this study is not attempting to do so. Rather, it is a correlational study of potential factors that can affect mental health. To play esports games in a healthy and appropriate manner that promotes personal psychological well-being, several recommendations are proposed in Table 5, based on the results of this study. This study is the first to examine the effects of playing esports games on psychological well-being, based on the gaming platform (PC gaming vs. mobile gaming), average daily gaming hours, and subjective attitudes of participants toward esports gaming, providing insights into the choice of indoor leisure activities and optimal patterns of esports gaming participation, especially during crises when outdoor events are restricted. Additionally, we believe that esports gaming holds the potential for integration into virtual reality exposure therapy in future studies, broadening its therapeutic applications.

**Table 5:** Practical suggestions of the present study

Main Suggestions
1. In the future, playing esports games is highly recommended as a safe, convenient, and inexpensive form of entertainment to cope with stress when staying indoors is encouraged or even mandatory to deal with particular situations.
2. For those who have no experience in esports games, it is preferable to try mobile esports games first. Compared with PC esports gaming, mobile esports gaming is less time-consuming and simpler to operate, making it relatively easier to create an entertaining environment.
3. During esports gaming, it is important to focus on whether we are truly immersed in the game, enjoying the experience rather than obsessing over winning or losing. This enjoyment is a key factor in benefiting from esports gaming, rather than just how long we play.
4. In competitive esports games, where rude and unfriendly behaviors such as verbal abuse often occur, we should avoid these behaviors, show positive attitudes, and respect other players by providing praise and encouragement while interacting.
5. Although longer gaming hours with positive attitudes may be beneficial in theory, it is important to prioritize daily responsibilities, especially for students. Additionally, long periods of sedentary behavior should be avoided when possible. Therefore, it is recommended to play esports games for no more than 2 to 3 hours per day to prevent excessive indulgence and internet addiction [38-42].

Despite its significant findings, the current study has several limitations. First, it is a cross-sectional study, and the questionnaire survey was not completed immediately following the most severe periods of the pandemic outbreak when staying at home was highly encouraged or even mandated. Thus, the time delay of survey completion may have influenced the accuracy of results because the mental states of participants could have changed due to potential personal or social factors implicated over the course of COVID-19. Second, the sample size was small, thereby limiting the generalizability of this study. Third, in consideration of various characteristics between general online video games with highlights on leisure and entertainment and esports games with highlights on competitiveness, high effort investment, and attention requirement, the impacts of non-esports games (e.g., single-player games) should be compared in further study. Fourth, while our hierarchical regression model aimed to minimize the impact of confounding factors (e.g., physical exercise habits), we acknowledge that a more rigorous analytical approach is necessary for future studies. Finally, this study was based on self-reported responses from young adults. Although the data were derived from an online professional data collection platform, more professional research methods in similar topics are needed to be carried out in the future when conditions permit.

## Conclusions

Esports gaming has become an increasingly popular alternative form of entertainment, particularly among young people. Playing esports games can be a beneficial way to foster psychological well-being, especially during times when people are required to stay indoors due to certain circumstances. However, inappropriate gaming patterns can result in negative outcomes, highlighting the importance of playing games in a positive and appropriate manner. This study indicates a correlation between positive attitudes toward esports gaming and enhanced psychological well-being among participants. However, further data are required to establish a causal relationship conclusively. Additionally, it may be beneficial for young people to monitor their daily gaming hours and engage with fellow players in a positive manner, as this could potentially contribute to improved psychological well-being, although further research is needed in this regard. In conclusion, esports gaming can serve as a valuable social outlet during the COVID-19 pandemic, providing opportunities for social interaction and positive effects on psychological well-being. However, more research is needed to fully understand the impact of gaming on mental health, particularly in relation to different types of games. It is important for individuals to approach gaming in a healthy and appropriate manner to promote personal well-being.

## Appendices

Appendix 1

Questionnaire Contents

Q1: What is your gender?

_____ Male; _____ Female

Q2: What is your age?

_____

Q3: What is your identity (occupation) (01/2020-06/2020)?

__Undergraduate; __Postgraduate; __Worker (Employee)

Q4: Did you have indoor exercise habit over 20 minutes each day (01/2020-06/2020)?

___Yes; ___No

Q5: Had you been a fan of esports games before the COVID-19 pandemic?

___Yes; ___No

Q6: Please choose the answer ‘Pink’.

___White; ___Yellow; ___Red; ___Black; ___Pink

Q7: What devices did you primarily play esports games on?

___PC (i.e., Personal Computer); ___Mobile

Q8: Also, which of the following esports games did you primarily play (01/2020-06/2020)?

___ League of Legends; ___ DOTA2; ___ CS: GO; ___ CrossFire; ___ Overwatch ___ PUBG; ___ Honor of Kings; ___ PUBG Mobile (i.e., Game for peace)

Q9: Please indicate the average amount of time you spend on video games each day (01/2020-06/2020).

__less than 1h; __1-3hrs; __3-5hrs; __5-7hrs; __over7hrs

Q10: During the period 01/2020-06/2020, did you experience any personal circumstances unrelated to gaming that caused a significant deterioration in your mental state? ___Yes; ___No

Q11: Please choose the answer ‘Apple’.

___Pear; ___Apple; ___Black; ___One; ___Number

Q12: Do you think playing esports games can positively improve psychological well-being during the pandemic (01/2020-06/2020)? ___Has potential negative impacts; Has no either positive or negative impact ___Be able to slightly improve; ___Be able to moderately improve; ___Be able to strongly improve

Appendix 2

Ryff’s Psychological Well-Being Scales (PWB), 42-Item Version

Please indicate your degree of agreement (using a score ranging from 1-6) to the following sentences.

1. I am not afraid to voice my opinions, even when they are in opposition to the opinions of most people.

2. In general, I feel I am in charge of the situation in which I live.

3. I am not interested in activities that will expand my horizons.

4. Most people see me as loving and affectionate.

5. I live life one day at a time and don't really think about the future.

6. When I look at the story of my life, I am pleased with how things have turned out.

7. My decisions are not usually influenced by what everyone else is doing.

8. The demands of everyday life often get me down.

9. I think it is important to have new experiences that challenge how you think about yourself and the world.

10. Maintaining close relationships has been difficult and frustrating for me.

11. I have a sense of direction and purpose in life.

12. In general, I feel confident and positive about myself.

13. I tend to worry about what other people think of me.

14. I do not fit very well with the people and the community around me.

15. When I think about it, I haven't really improved much as a person over the years.

16. I often feel lonely because I have few close friends with whom to share my concerns.

17. My daily activities often seem trivial and unimportant to me.

18. I feel like many of the people I know have gotten more out of life than I have.

19. I tend to be influenced by people with strong opinions.

20. I am quite good at managing the many responsibilities of my daily life.

21. I have the sense that I have developed a lot as a person over time.

22. I enjoy personal and mutual conversations with family members or friends.

23. I don't have a good sense of what it is I'm trying to accomplish in life.

24. I like most aspects of my personality.

25. I have confidence in my opinions, even if they are contrary to the general consensus.

26. I often feel overwhelmed by my responsibilities

27. I do not enjoy being in new situations that require me to change my old familiar ways of doing things.

28. People would describe me as a giving person, willing to share my time with others.

29. I enjoy making plans for the future and working to make them a reality.

30. In many ways, I feel disappointed about my achievements in life.

31. It's difficult for me to voice my own opinions on controversial matters.

32. I have difficulty arranging my life in a way that is satisfying to me.

33. For me, life has been a continuous process of learning, changing, and growth.

34. I have not experienced many warm and trusting relationships with others.

35. Some people wander aimlessly through life, but I am not one of them.

36. My attitude about myself is probably not as positive as most people feel about themselves.

37. I judge myself by what I think is important, not by the values of what others think is important.

38. I have been able to build a home and a lifestyle for myself that is much to my liking.

39. I gave up trying to make big improvements or changes in my life a long time ago.

40. I know that I can trust my friends, and they know they can trust me.

41. I sometimes feel as if I've done all there is to do in life.

42. When I compare myself to friends and acquaintances, it makes me feel good about who I am.

Scoring Instruction

1) Recode negative phrased items: # 3, 5, 10, 13, 14, 15, 16, 17, 18, 19, 23, 26, 27, 30, 31, 32, 34, 36, 39, 41. (i.e., if the scored is 6 in one of these items, the adjusted score is 1; if 5, the adjusted score is 2 and so on...)

2) Add together the final degree of agreement in the 6 dimensions:

Autonomy: items 1,7,13,19,25, 31, 37

Environmental mastery: items 2,8,14,20,26,32,38

Personal Growth: items 3,9,15,21,27,33,39

Positive Relations: items: 4,10,16,22,28,34,40

Purpose in life: items: 5,11,17,23,29,35,41

Self-acceptance: items 6,12,18,24,30,36,42
